# Association Between Insulin Therapy and In‐Hospital Death in Chronic Heart Failure Patients With Type 2 Diabetes Mellitus: Mediated by Plasma Volume

**DOI:** 10.1111/1753-0407.70172

**Published:** 2025-11-30

**Authors:** Xiaofei Luo, Lin Zhang, Boxuan Feng, Xiangqin Ou, Jingyi Lin, Danping Zhuo, Haohao Gao, Li Liu, Minghao Xu, Jialing Liu, Fan Jia, Guanwei Fan

**Affiliations:** ^1^ First Teaching Hospital of Tianjin University of Traditional Chinese Medicine National Clinical Research Center for Chinese Medicine Acupuncture and Moxibustion Tianjin China; ^2^ Tianjin University of Traditional Chinese Medicine Tianjin China; ^3^ Tianjin Academy of Traditional Chinese Medicine Affiliated Hospital Tianjin China; ^4^ Department of Biostatistics and Data Science The University of Texas Health Science Center at Houston School of Public Health Houston Texas USA

**Keywords:** chronic heart failure, in‐hospital death, insulin, type 2 diabetes mellitus

## Abstract

**Aims:**

To examine the association of insulin therapy with in‐hospital death and whether this association is mediated by elevated plasma volume among chronic heart failure (CHF) patients with type 2 diabetes mellitus (T2DM).

**Materials and Methods:**

A retrospective study combining two electronic medical records: the Medical Information Mart for Intensive Care (MIMIC) database and the Tianjin Heart Failure with Integrated Treatment (TJHFIT) database. Propensity score matching (1:2 ratio) was conducted in CHF‐T2DM patients to eliminate the differences in demographics, comorbidity, and the severity of diabetes. Then, conditional logistic regression, restricted cubic spline (RCS) modeling, and mediation analysis were processed.

**Results:**

A total of 7997 CHF‐T2DM patients were included, with 6112 from MIMIC (2241 received insulin therapy and 3871 not), and 1885 from TJHFIT (911 received insulin therapy and 974 not). Multivariable conditional logistic regression revealed a significant association between insulin therapy during hospitalization and in‐hospital death in both cohorts (MIMIC: OR, 1.37 [95% CI, 1.14–1.63]; TJHFIT: OR, 2.56 [95% CI, 1.53–4.27]). Insulin therapy was associated with a higher likelihood of ΔePVS > 0 (MIMIC: OR, 1.11 [95% CI, 1.02–1.28]; TJHFIT: OR, 1.37 [95% CI, 1.13–1.66]). RCS revealed both ePVS and ΔePVS were associated with in‐hospital death in a nonlinear fashion. The ePVS at discharge mediated insulin‐associated in‐hospital death with a proportion of 12.0% (MIMIC) and 13.2% (TJHFIT).

**Conclusions:**

Insulin therapy was associated with elevated odds of in‐hospital death among CHF‐T2DM patients, which was mediated by plasma volume.

**Trial Registration:**

The research has been registered (ChiCTR2300077220)

## Summary

The study demonstrates that insulin use was associated with increased in‐hospital death in patients with CHF‐T2DM, which is mediated by increased plasma volume.

## Graphical abstract

This retrospective analysis of two independent cohorts (MIMIC and TJHFIT) in patients with comorbid chronic heart failure and type 2 diabetes mellitus (CHF‐T2DM) investigates the association between in‐hospital insulin therapy and mortality. After adjustment for confounders, insulin therapy was significantly associated with increased odds of in‐hospital death. Analysis further demonstrated that this association was partly mediated by an increase in estimated plasma volume, which itself was nonlinearly associated with mortality, explaining approximately 12%–13% of the elevated risk.

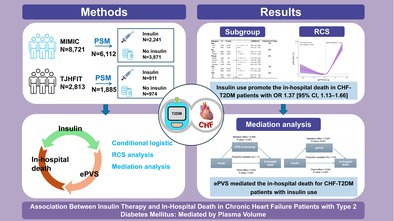

**Abbreviations**
ACEI/ARB: angiotensin‐converting enzyme inhibitors/angiotensin II receptor antagonists
ACME: average causal mediation effect
ADE: average direct effect
AF: atrial fibrillation
CAD: coronary artery disease
CHF: chronic heart failure
CKD: chronic kidney disease
EMRs: electronic medical records
ePVS: estimated plasma volume status
FPG: fasting plasma glucose
HFmrEF: heart failure with mildly reduced ejection fraction
HFpEF: heart failure with preserved ejection fraction
HFrEF: heart failure with reduced ejection fraction
ICD: International Classification of Diseases
LOS: length of hospital stay
MIMIC: Medical Information Mart for Intensive Care
OR: odd ratio
PSM: propensity score matching
PV: plasma volume
RCS: restricted cubic spline
SMD: standardized mean differences
T2DM: type 2 diabetes mellitus
TJHFIT: Tianjin Heart Failure with Integrated Treatment

## 1 Introduction

Type 2 diabetes mellitus (T2DM) was a common comorbidity and an independent risk factor for chronic heart failure (CHF) [1, 2, 3, 4]. The 2023 ESC Guidelines mentioned insulin as one of the major schemes for blood glucose management among CHF‐T2DM patients [5], yet many observational and clinical trial studies yielded contrasting conclusions regarding the consequence for insulin therapy receivers in CHF patients [6, 7, 8, 9, 10], due to differences in cardiac function across populations. Hence, investigating the impact of insulin therapy on CHF‐T2DM patients is imperative for real‐world clinical practice.

Changes of plasma volume (PV) in response to insulin therapy might play a crucial role in disease progression. Physiologically, insulin injection stimulates renal tubular sodium chloride reabsorption, exacerbating fluid retention in the body [11, 12]. This mechanism may lead to an increase in PV that results in an increase in ventricular preload [13, 14, 15], leading to a poor prognosis for CHF patients [16]. The gold standard methods (radio‐isotope‐labeled albumin [14] and Evans blue dye dilution) to measure PV, however, were invasive and posed an additional risk for physiologically compromised patients [17, 18]. As an alternative, the estimated plasma volume status (ePVS) calculated as a hemoglobin/hematocrit‐based indirect formula allows for a noninvasive, instantaneous, convenient calculation of PV [19, 20, 21].

Several knowledge gaps exist in the relationship between insulin therapy, PV, and in‐hospital death of CHF‐T2DM patients. First, the evidence of the association between insulin therapy and in‐hospital death was still limited in CHF patients. Second, it remained unclear whether insulin therapy could have an impact on PV, which might further lead to a poor prognosis. Third, whether this association was consistent across varying CHF subtypes remains unknown.

Our study aims to address these gaps by examining the association of insulin therapy with in‐hospital death and the potential mediating role of PV changes in CHF‐T2DM patients.

## 2 Methods

### 2.1 Study Populations

This study included two electronic medical records (EMRs), the Medical Information Mart for Intensive Care (MIMIC) database [22] and the Tianjin Heart Failure with Integrated Treatment (TJHFIT). MIMIC‐IV authorized the extraction of data for the study (ID: 55394138), and the participants were collected from 2008 to 2019. TJHFIT was registered (ChiCTR2300077220) to explore the therapeutic effects of traditional Chinese and Western medicine in treating heart failure with preserved ejection fraction (HFpEF) in the Tianjin region. The participants were enrolled from 2006 to 2018, from 43 hospitals in Tianjin, China.

The study population was selected following the inclusion criteria: (1) age at hospital admission ≥ 18 years; (2) hospitalization duration > 24 h; (3) confirmed diagnoses of both CHF and T2DM at admission. Patients lacking baseline hematocrit, hemoglobin, and fasting plasma glucose (FPG) measurements were excluded to achieve a complete case analysis. A total of 8712 and 2813 patients were identified from MIMIC and TJHFIT.

### 2.2 Propensity Scores

**FIGURE 1:**
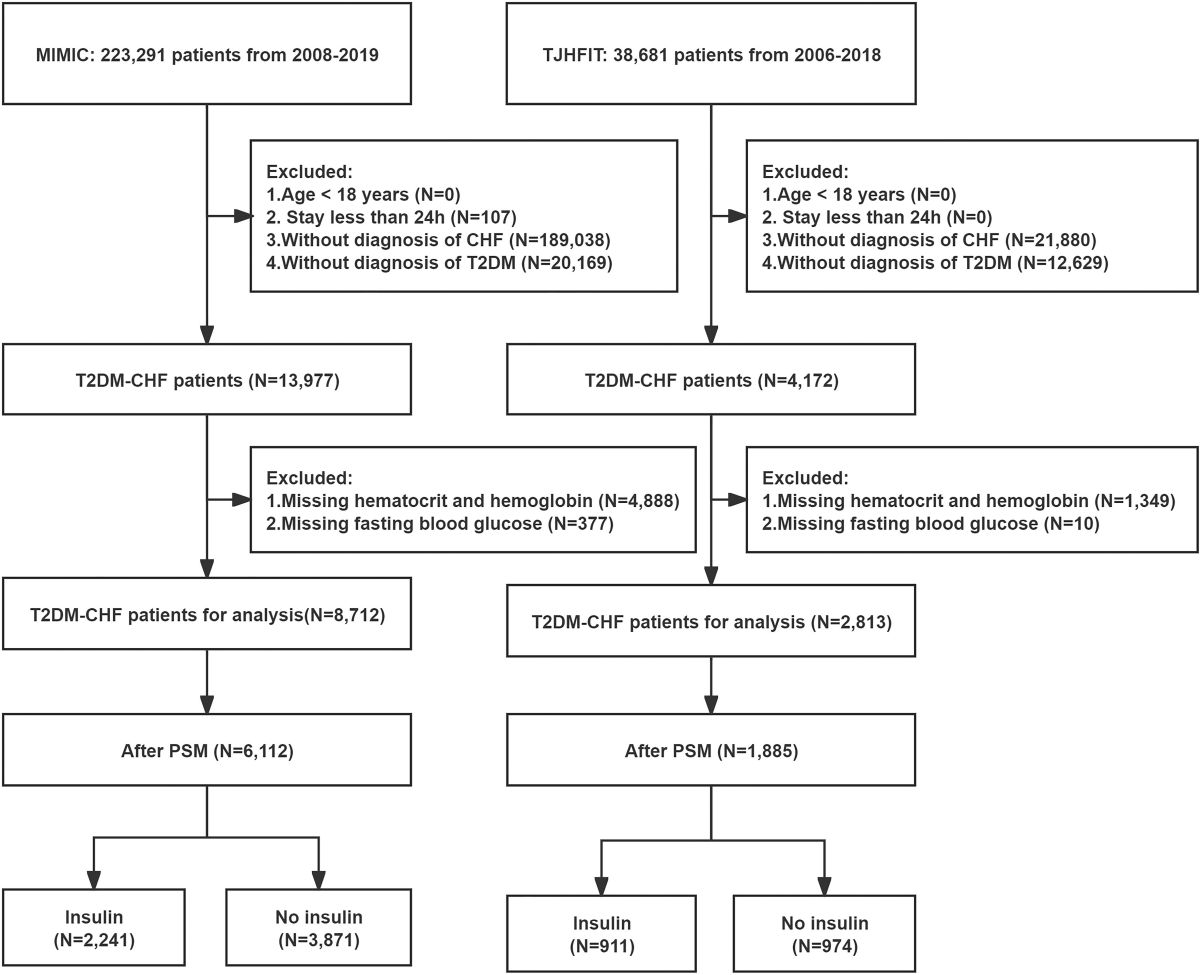
Flow diagram that describes the study populations.

Propensity score matching (PSM) was performed to minimize potential biases [23]. The propensity scores were estimated using a logistic regression model and included baseline demographic (age and sex), comorbidities (e.g., hypertension, hyperlipidemia), and laboratory tests (FPG and ePVS at admission) as covariates. The matching was performed between insulin therapy receivers and non‐receivers using a 1:2 nearest‐neighbor algorithm without replacement, with a caliper width set to 0.02 times the standard deviation of the logit‐transformed propensity score to minimize bias. The balance of covariates between the matched groups was assessed using standardized mean differences (SMD), with an SMD < 0.1 considered a good balance of covariates (Table S1). We included a flow diagram to show the process of subject inclusions, exclusions, and PSM (Figure 1). A total of 2241 insulin therapy receivers (vs. 3871 non‐receivers) from MIMIC and 911 insulin therapy receivers (vs. 974 non‐receivers) from TJHFIT were included in the final cohort.

### 2.3 Definitions and Classifications of CHF‐T2DM Patients

Diagnosis of T2DM and CHF depends on the International Classification of Diseases (ICD) codes and clinical features (Tables S2 and S3). According to the 2022 AHA/ACC/HFSA Guideline [2], we classified CHF patients based on the left ventricular ejection fraction (LVEF) value: HFpEF was defined by LVEF ≥ 50%; heart failure with mildly reduced ejection fraction (HFmrEF) was defined by LVEF between 40% and 50%; heart failure with reduced ejection fraction (HFrEF) was defined by LVEF ≤ 40%. Patients without LVEF records were classified as “Unknown.”

### 2.4 Insulin Therapy

Patients who received insulin therapy during their hospital stay, according to the EMRs, were considered insulin therapy recipients.

### 2.5 ePVS and ΔePVS

Duarte's formula indicated the instantaneous PV [24] with: $\text{ePVS}=\frac{1-\text{hematocrit}}{\text{hemoglobin}\;\left(g/\mathrm{dL}\right)}\times 0.01$ (mL/g). The ΔePVS was defined as the difference in ePVS at admission and discharge.

### 2.6 Outcomes

In‐hospital death was derived from records in EMRs as the main outcome measure. The secondary outcome was ΔePVS, calculated from formula 2.5 above.

### 2.7 Covariates

The study retrieved demographics, comorbidities, medication history, and laboratory tests from EMRs. Demographic information included age and sex. Comorbidities included coronary artery disease (CAD), hypertension, hyperlipidemia, chronic kidney disease (CKD), atrial fibrillation (AF), and anemia. Medications were sorted into conventional CHF medication [25, 26] and common oral hypoglycemic agents [27]. CHF medications involve three drug types: angiotensin‐converting enzyme inhibitors (ACEI)/angiotensin II receptor antagonists (ARB), beta‐blockers, and aldosterone receptor antagonists. Common oral hypoglycemic agents include biguanides and sulfonylureas. Study participants were classified as users and nonusers by the drug categories mentioned above. The laboratory tests of hematocrit, hemoglobin, and FPG, examined from EMRs, were included in the study. None of these laboratory tests had missing values after the exclusion of subjects in the study. As the secondary outcome indicator, we categorized the patients of ΔePVS by the value above or below 0. The ΔePVS and ePVS at discharge were considered for mediation analyses.

### 2.8 Statistical Analysis

All statistical analyses were conducted using R (version 4.3.2). For continuous variables, the normality assumption was first evaluated. A Kolmogorov–Smirnov test was used to examine whether continuous variables are normally distributed. All continuous variables were not normally distributed and were expressed as median values with interquartile ranges. Non‐normally distributed continuous variables were analyzed using the Mann–Whitney *U* test. Categorical variables were expressed as frequency and percentage and were compared via the chi‐square test.

Conditional logistic regression was performed to assess the association between insulin therapy and in‐hospital death of CHF‐T2DM patients, mediated by ΔePVS. Model 0 provided the unadjusted results. Covariate‐adjusted models were constructed to control for confounding. Model 1 included age and sex. Model 2 included Model 1 covariates plus length of hospital stay (LOS), CAD, hypertension, CKD, and AF. Model 3 included Model 2 covariates plus ACEI, ARB, beta‐blockers, aldosterone receptor antagonists, furosemide, biguanides, and sulfonylureas.

Subgroup analyses were carried out to detect potential effect modifications by age (younger than 60 years or 60 years or older), sex (male and female), HF subtypes (HFpEF, HFmrEF, HFrEF, and unknown), CAD (CAD vs. no CAD), and CKD (CKD vs. no CKD). Given the insufficient sample size in subgroups, logistic regression was employed for subgroup analyses to mitigate overfitting risks associated with multivariable modeling.

A restricted cubic spline (RCS) was performed to explore the potential nonlinear relationship between ePVS (ΔePVS) and in‐hospital death and to identify the threshold associated with odds greater than 0. The RCS results were presented after the Model 3 adjustments.

To test the hypothesis that insulin increases the odds of in‐hospital death in CHF‐T2DM patients by increasing PV, the mediation analysis was conducted using the *mediation* R package. The ePVS at discharge and ΔePVS were considered the mediators of all previously mentioned covariate adjustments. Each mediation model included the same covariates as Model 3. The analysis employed a quasi‐Bayesian Monte Carlo simulation approach (*n* = 1000 simulations) to estimate the average causal mediation effect (ACME), average direct effect (ADE), and total effect, along with their 95% confidence intervals (CIs).

## 3 Results

### 3.1 Baseline Characteristics

In both cohorts, consistent medication pattern differences were observed between insulin and non‐insulin patients (Table 1).

**TABLE 1:** Baseline patient characteristics after propensity score matching on insulin therapy.

Baseline characteristics	MIMIC				TJHFIT			
	Overall	Insulin	No insulin	*p*	Overall	Insulin	No insulin	*p*
Number of patients	6112	2241	3871		1885	911	974	
Demographics								
Age, years (median [IQR])	71 (62, 79)	70 (62, 78)	71 (62, 80)	0.01	71 (64, 78)	70 (63, 78)	71 (64, 78)	0.3
Female, *n* (%)	2492 (41)	909 (41)	1583 (41)	0.8	946 (50)	443 (49)	503 (52)	0.2
Smoked, *n* (%)	2465 (40)	907 (40)	1558 (40)	0.9	568 (30)	283 (31)	285 (29)	0.4
Comorbidities								
CAD, *n* (%)	4083 (67)	1513 (68)	2570 (66)	0.4	1547 (82)	750 (82)	797 (82)	0.8
Hypertension, *n* (%)	1939 (32)	698 (31)	1241 (32)	0.5	1575 (84)	766 (84)	809 (83)	0.5
Hyperlipidemia, *n* (%)	3725 (61)	1368 (61)	2357 (61)	> 0.9	425 (23)	200 (22)	225 (23)	0.6
CKD, *n* (%)	4370 (71)	1628 (73)	2742 (71)	0.13	1476 (78)	835 (76)	780 (80)	0.05
AF, *n* (%)	2631 (43)	955 (43)	1676 (43)	0.6	383 (20)	185 (20)	198 (20)	> 0.9
Anemia, *n* (%)	3068 (50)	1162 (52)	1906 (49)	0.49	494 (26)	255 (28)	239 (25)	0.09
LOS, days (median [IQR])	7 (4, 12)	8 (4, 13)	7 (4, 11)	0.3	14 (11, 17)	14 (10, 18)	14 (11, 17)	0.8
HF subtypes				< 0.01				0.2
HFpEF, *n* (%)	1399 (23)	563 (25)	836 (22)		388 (21)	177 (19)	211 (22)	
HFmrEF, *n* (%)	254 (4.2)	96 (4.3)	158 (4.1)		128 (6.8)	61 (6.7)	67 (6.9)	
HFrEF, *n* (%)	1105 (18)	434 (19)	671 (17)		237 (13)	128 (14)	109 (11)	
Unknown, no. (%)	3354 (55)	1148 (51)	2206 (57)		1132 (60)	545 (60)	587 (60)	
Laboratory tests								
Hematocrit, % (median [IQR])	34 (29, 38)	34 (29, 38)	34 (29, 38)	0.5	33 (20, 39)	33 (24, 38)	33 (14, 39)	0.2
Hemoglobin, median (IQR), g/dL	10.8 (9.3, 12.3)	10.8 (9.3, 12.3)	10.8 (9.3, 12.4)	> 0.9	12.1 (10.1, 13.5)	11.9 (9.9, 13.4)	12.2 (10.3, 13.6)	0.02
FPG at admission, mg/dL (median [IQR])	160 (119, 217)	167 (120, 229)	157 (118, 213)	< 0.01	144 (113, 187)	145 (111, 190)	142 (113, 181)	0.6
FPG at discharge, mg/dL (median [IQR])	130 (102, 169)	132 (102, 174)	129 (102, 167)	0.1	123 (100, 157)	131 (105, 172)	117 (97, 142)	< 0.01
ePVS at admission, mL/g (median [IQR])	6.14 (5.02, 7.60)	6.13 (5.05, 7.56)	6.14 (5.00, 7.61)	0.8	6.15 (4.76, 7.90)	6.11 (4.78, 7.91)	6.21 (4.71, 7.90)	> 0.9
ePVS at discharge, mL/g (median [IQR])	7.14 (5.82, 8.47)	7.29 (6.17, 8.59)	7.01 (5.66, 8.40)	< 0.01	5.63 (4.65, 7.32)	5.82 (4.75, 7.55)	5.42 (4.53, 7.08)	< 0.01
ΔePVS, mL/g (median [IQR])	0.61 (−0.14, 1.57)	0.75 (−0.12, 1.99)	0.55 (−0.15, 1.39)	< 0.01	0.00 (−1.49, 0.70)	0.12 (−1.05, 0.80)	−0.06 (−2.11, 0.56)	< 0.01
Medication histories								
ACEI, *n* (%)	1902 (31)	692 (31)	1210 (31)	0.8	351 (19)	168 (18)	183 (19)	0.8
ARB, *n* (%)	812 (13)	295 (13)	517 (13)	0.8	656 (35)	321 (35)	335 (34)	0.7
Beta‐blockers, *n* (%)	4744 (78)	1800 (80)	2944 (76)	< 0.01	1002 (53)	474 (52)	528 (54)	0.3
*n* (%)	608 (9.9)	188 (8.4)	420 (11)	< 0.01	794 (42)	395 (43)	399 (41)	0.3
Furosemide, *n* (%)	4289 (70)	1687 (75)	2602 (67)	< 0.01	1034 (55)	551 (60)	483 (50)	< 0.01
Biguanides, *n* (%)	427 (7.0)	214 (9.5)	213 (5.5)	< 0.01	402 (21)	181 (20)	221 (23)	0.14
Sulfonylureas, *n* (%)	284 (4.6)	123 (5.5)	161 (4.2)	0.02	238 (13)	104 (11)	134 (14)	0.13

Abbreviations: ACEI, angiotensin‐converting enzyme inhibitors; AF, atrial fibrillation; ARB, angiotensin II receptor antagonists; CAD, coronary artery disease; CKD, chronic kidney disease; ePVS, estimated plasma volume status; FPG, fasting plasma glucose; HFmrEF, heart failure with mildly reduced ejection fraction; HFpEF, heart failure with preserved ejection fraction; HFrEF, heart failure with reduced ejection fraction; IQR, interquartile range; ΔePVS, the difference in ePVS at admission and discharge.

In MIMIC, 2241 patients received insulin therapy, with a median age of 71 years (IQR 62–79), 41% female, and 40% with a smoking history. Significant differences across HF subtypes were found (*p* < 0.01), with higher HFpEF prevalence (25% vs. 22%) found in the insulin group. Laboratory tests revealed a higher FPG at admission (median 167 vs. 157 mg/dL, *p* < 0.01) and a higher ePVS at discharge (median 7.29 vs. 7.01 mL/g, *p* < 0.01) in the insulin than in the no‐insulin group. More beta‐blockers (80% vs. 76%, *p* < 0.01), furosemide (75% vs. 67%, *p* < 0.01), and biguanides (9.5% vs. 5.5%, *p* < 0.01) were used in the insulin group.

In TJHFIT, 911 patients received insulin, with a median age of 71 years (IQR 64–78), 50% were male, and 30% were smokers. HF subtypes were similar between the two groups (*p* = 0.2). The insulin group had lower hemoglobin levels (median 11.9 vs. 12.2 g/dL, *p* = 0.02) and higher FPG at discharge (median 131 vs. 117 mg/dL, *p* < 0.01), higher ΔePVS (median 0.12 vs. −0.06 mL/g, *p* < 0.01) and higher ePVS at discharge (median 5.82 vs. 5.42 mL/g, *p* < 0.01) than non‐receivers. Furosemide was more frequently used in insulin therapy receivers (60% vs. 50%, *p* < 0.01).

The MIMIC and TJHFIT have different population characteristics. MIMIC had a lower proportion of insulin‐treated patients (36.7% vs. 48.3%), with higher male predominance (59% vs. 50%) and higher smoking rates (40% vs. 30%). TJHFIT patients exhibited a heavier comorbidity burden, including higher rates of CAD (82% vs. 67%), hypertension (84% vs. 32%), and CKD (78% vs. 71%). MIMIC had higher FPG at admission (median 160 vs. 144 mg/dL), while the TJHFIT insulin group achieved better FPG at discharge (median 131 vs. 117 mg/dL). Medication patterns differed between the two cohorts, with beta‐blockers being more commonly used in MIMIC (78% vs. 53%) and aldosterone receptor antagonists being more commonly used in TJHFIT (42% vs. 9.9%). These differences reflected the variations in regional practices.

### 3.2 Logistic Regressions

#### 3.2.1 Insulin Therapy and In‐Hospital Death

Multivariable conditional logistic regression was performed to assess the association between insulin therapy and in‐hospital death in both cohorts using four progressively adjusted models (Table 2). Insulin therapy was consistently associated with higher odds of in‐hospital death across all models. In MIMIC, the adjusted matched odds ratio (OR) increased from 1.39 (95% CI, 1.20–1.61; *p* < 0.01) in Model 0 to 1.37 (95% CI, 1.14–1.63; *p* < 0.01) in Model 3. In TJHFIT, the aOR remained stable across models, with values of 2.15 (95% CI, 1.50–3.09; *p* < 0.01) in Model 0 and 2.56 (95% CI, 1.53–4.27; *p* < 0.01) in Model 3. These consistent findings across all model adjustments for both cohorts underscore the significance that insulin therapy is associated with in‐hospital death.

**TABLE 2:** The mediation analysis of association between insulin therapy, ΔePVS, and in‐hospital death.

Model	Outcome	MIMIC		TJHFIT	
		OR (95% CI)	*p*	OR (95% CI)	*p*
Model 0	In‐hospital death	1.39 (1.20, 1.61)	< 0.01	2.15 (1.50, 3.09)	< 0.01
	ΔePVS > 0	1.10 (1.01, 1.27)	0.04	1.29 (1.08, 1.55)	0.01
Model 1	In‐hospital death	1.36 (1.17, 1.59)	< 0.01	2.25 (1.53, 3.31)	< 0.01
	ΔePVS > 0	1.10 (1.01, 1.27)	0.03	1.30 (1.08, 1.57)	< 0.01
Model 2	In‐hospital death	1.34 (1.14, 1.58)	< 0.01	2.44 (1.59, 3.74)	< 0.01
	ΔePVS > 0	1.10 (1.01, 1.27)	0.03	1.30 (1.08, 1.56)	0.01
Model 3	In‐hospital death	1.37 (1.14, 1.63)	< 0.01	2.56 (1.53, 4.27)	< 0.01
	ΔePVS > 0	1.11 (1.02, 1.28)	0.04	1.37 (1.13, 1.66)	< 0.01

*Note:* The primary adjusted methods were: Model 0: included no covariate; Model 1 includes demographic information (age and sex); Model 2 includes all covariates from Model 1 plus length of hospital stay, CAD, hypertension, CKD, and AF; Model 3 includes all covariates from Model 2 plus ACEI, ARB, Beta‐blockers, Aldosterone receptor antagonists, Furosemide, Biguanides, and Sulfonylureas.

Abbreviations: ACEI, angiotensin‐converting enzyme inhibitors; AF, atrial fibrillation; ARB, angiotensin II receptor antagonists; CAD, coronary artery disease; CKD, chronic kidney disease.

Subgroup analyses were performed and stratified (Tables S4 and S5) by age, sex, HF subtypes, CAD, and CKD. The association between insulin therapy receivers and in‐hospital death was stronger among CKD patients for MIMIC (OR, 1.66 [95% CI, 1.41–1.69], *p* < 0.01, *p* for interaction = 0.01) and TJHFIT (OR, 2.94 [95% CI, 1.86–4.75], *p* < 0.01, *p* for interaction = 0.01). In contrast, the association between insulin and in‐hospital death did not differ significantly between age, sex, HF subtypes, and CAD in both cohorts (all *p* for interaction > 0.05).

Insulin therapy was significantly associated with increased odds of in‐hospital death across all age groups in MIMIC (aged > 60 years: OR, 1.43 [95% CI, 1.21–1.70], *p* < 0.01; aged ≤ 60 years: OR, 1.99 [95% CI, 1.35–2.95], *p* < 0.01). However, the same phenomenon was only observed in patients aged > 60 years in TJHFIT (aged > 60 years: OR, 2.21 [95% CI, 1.50–3.30], *p* < 0.01), not those younger than 60 (aged ≤ 60 years: OR, 2.34 [95% CI, 0.48–13.3], *p* = 0.3). In MIMIC, HFpEF (MIMIC: OR, 1.79 [95% CI, 1.26–2.54]) demonstrated a stronger association compared to other HF subtypes, and similar trends were found for TJHFIT (TJHFIT: OR, 3.61 [95% CI, 1.43–9.99]).

#### 3.2.2 Insulin Therapy and ΔePVS

The association between insulin therapy and ΔePVS was positive across MIMIC and TJHFIT, with consistent effect sizes from Model 0 to Model 3 (Table 2).

### 3.3 ΔePVS and In‐Hospital Death

**FIGURE 2:**
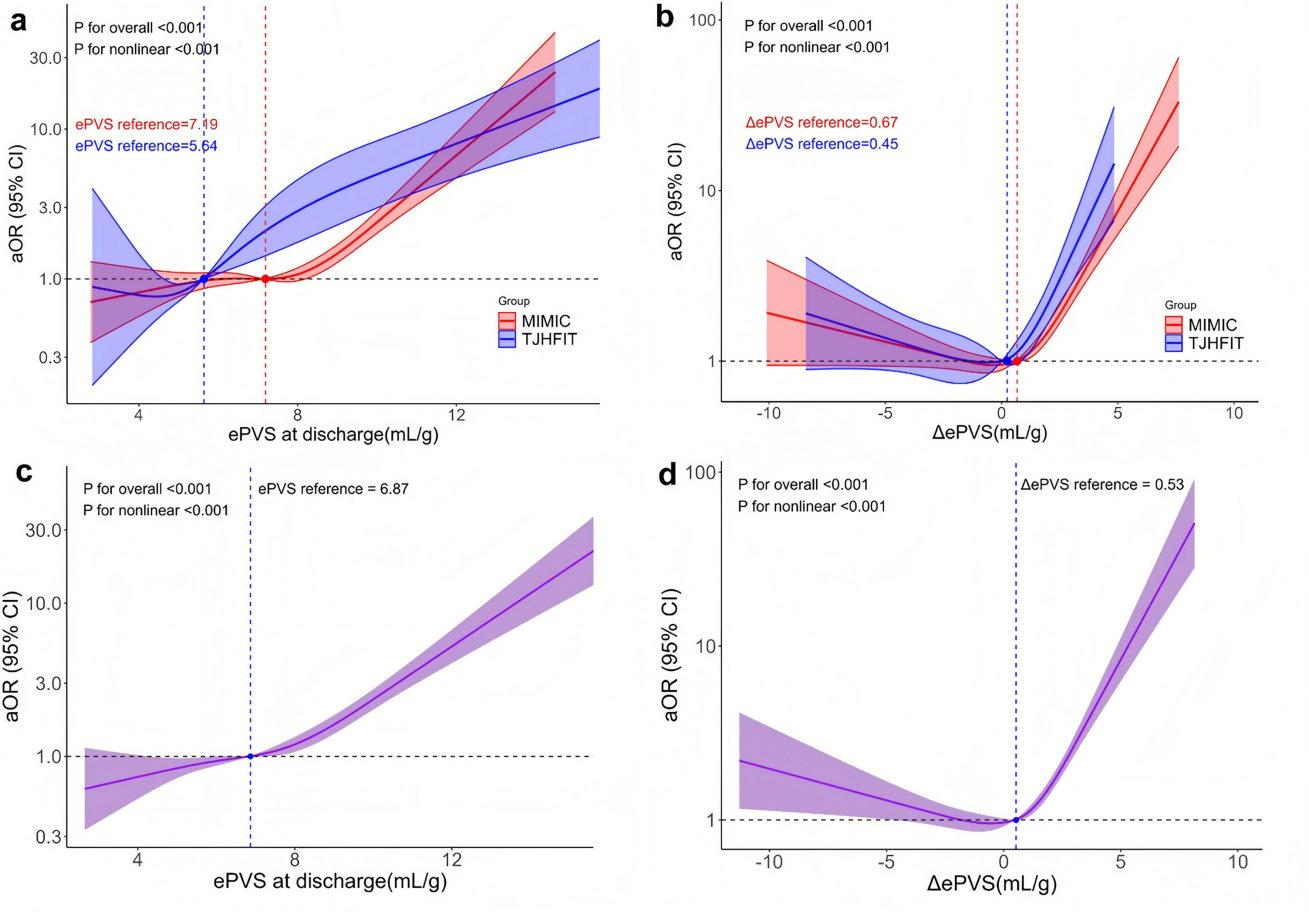
Panel (a) describes the RCS curve plot of ePVS at discharge separated by two cohorts. Panel (b) describes the RCS curve plot of ΔePVS separated by two cohorts. Panel (c) describes the RCS curve plot of ePVS at discharge for all people by two cohorts. Panel (d) describes the RCS curve plot of ΔePVS for all people by two cohorts. The primary adjusted methods were: Model 3 includes age, sex, length of hospital stay, CAD, hypertension, CKD, AF, ACEI, ARB, beta‐blockers, aldosterone receptor antagonists, furosemide, biguanides, and sulfonylureas. ACEI, angiotensin‐converting enzyme inhibitors; AF, atrial fibrillation; ARB, angiotensin II receptor antagonists; CAD, coronary artery disease; CKD, chronic kidney disease; ePVS, estimated plasma volume status; ΔePVS the difference in ePVS at admission and discharge.

RCS analyses revealed consistent nonlinear associations of in‐hospital death with ePVS at discharge and ΔePVS in two cohorts (Figure 2) after model 3 adjustment for demographic variables, comorbidities, and pharmacotherapies. For both ePVS at discharge and ΔePVS, the curves are characterized by a J‐shaped pattern, where the odds were greater than 1 marked at specific inflection points. The clinical impact of ePVS and ΔePVS is not uniformly distributed but concentrated within distinct ranges.

### 3.4 Mediation Analyses

**FIGURE 3:**
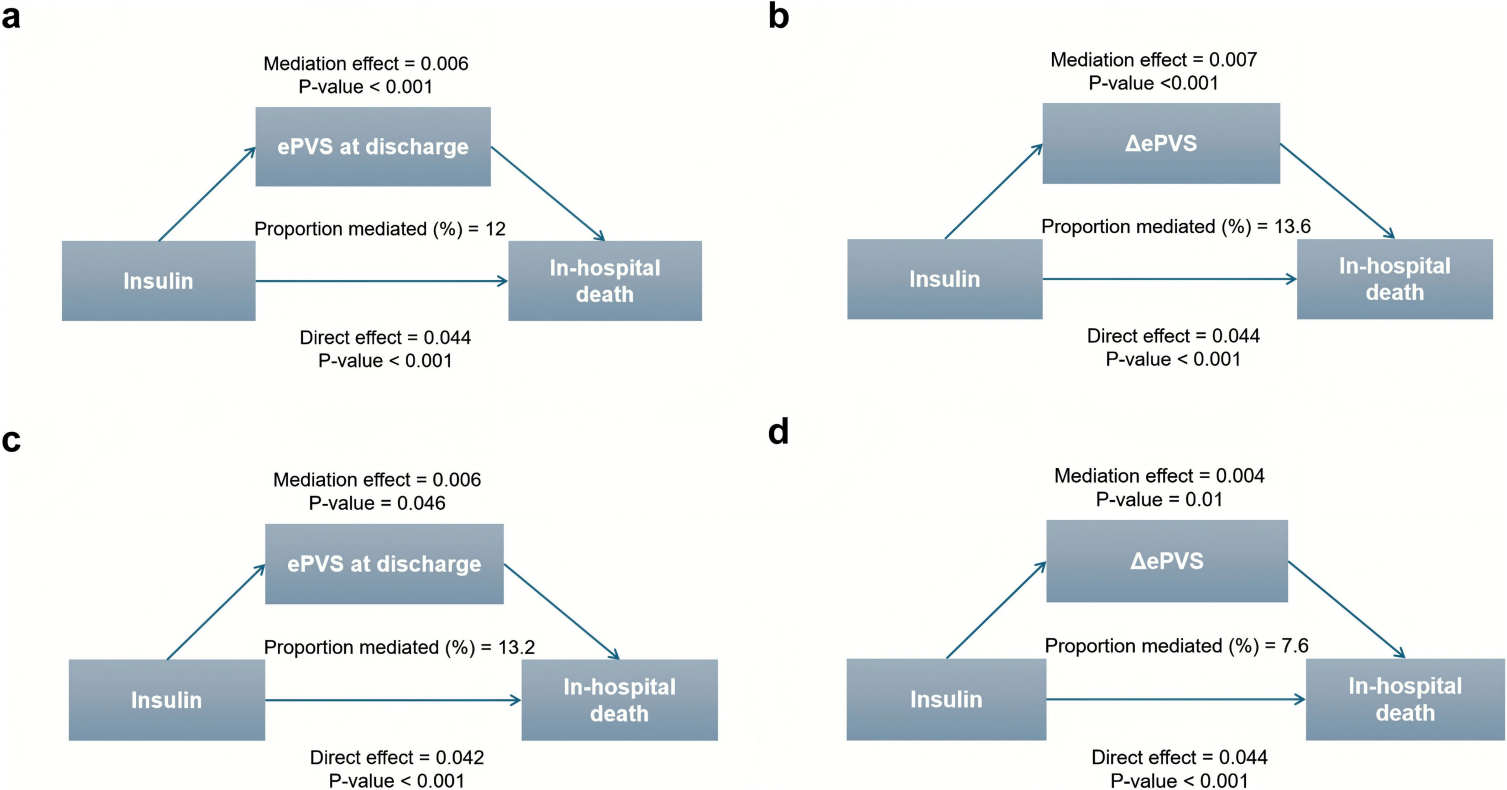
Panels (a, b) represent the mediation analysis in MIMIC, while panels (c, d) represent the mediation analysis in TJHFIT. The primary adjusted methods were: Model 3 includes age, sex, length of hospital stay, CAD, hypertension, CKD, AF, ACEI, ARB, beta‐blockers, aldosterone receptor antagonists, furosemide, biguanides, and sulfonylureas. ACEI, angiotensin‐converting enzyme inhibitors; AF, atrial fibrillation; ARB, angiotensin II receptor antagonists; CAD, coronary artery disease; CKD, chronic kidney disease; ePVS, estimated plasma volume status; ΔePVS the difference in ePVS at admission and discharge.

Mediation on ePVS was investigated for the association between insulin therapy and in‐hospital death (Figure 3 and Table S6). In MIMIC, ePVS at discharge mediated 12% (95% CI, 7%–20%; *p* < 0.05) of the total effect, while ΔePVS accounted for 13.6% (95% CI, 7.8%–21.6%; *p* < 0.05). The TJHFIT cohort demonstrated comparable patterns, with ePVS at discharge mediating 13.2% (95% CI, 0.4%–30.2%; *p* < 0.05) and ΔePVS mediating 7.6% (95% CI, 1.5%–20.3%; *p* < 0.05) of the total effect. All direct effects on ΔePVS and ePVS at discharge remained statistically significant positive (*p* < 0.05), indicating that insulin use is associated with high and increased PV which is associated with elevated in‐hospital death.

## 4 Discussion

This study indicates that insulin therapy is associated with higher odds of in‐hospital death in CHF‐T2DM patients, mediated by PV expansion during hospitalization, suggesting a plausible mechanistic pathway. These findings were consistent across two distinct cohorts in the US and China, reinforcing their generalizability.

The debate over insulin therapy might be explained by cardiac function differences across study populations. In general, insulin should be protective against adverse outcomes for T2DM patients with no cardiac morbidity. Real‐world data have found that in patients diagnosed with T2DM for less than 6 months, insulin therapy reduced the risk of stroke and hospitalization for heart failure [6]. From the results of the ORIGIN trial [7] and the meta‐analysis on cardiovascular incidence in T2DM patients [28, 29], insulin use showed no significant association with increasing stroke incidence or cardiovascular death in patients with T2DM. In contrast, T2DM patients with cardiac insufficiency had poorer outcomes after insulin use. According to the secondary analysis of several clinical trials [9, 10], insulin therapy increased hospitalization, cardiovascular disease death, and all‐cause mortality for T2DM patients with heart failure. For T2DM patients with milder cardiovascular diseases such as AF, insulin use increased the risk of HF events, cardiovascular and all‐cause mortality [30]. Together with existing evidence, our findings suggest that normal cardiac function may be crucial for CHF‐T2DM patients to receive insulin.

Mediation analysis suggested that increasing PV might be the cause of insulin‐induced in‐hospital death in CHF‐T2DM patients. Myocardial tissue with functional insufficiency is less tolerant to increased [31] and hypoglycemia [32]. The importance of PV overload for heart failure was highlighted in physiology [33] and as a predictor [34, 35] or stratifier [36] in human studies, which remain stable across compensated [37] and decompensated heart failure [24]. Notably, we found in our study that CKD patients have higher insulin‐associated in‐hospital death compared to non‐CKD patients. This can be explained by elevated PV being associated with all‐cause mortality in hemodialysis patients [38] and non‐dialysis CKD [39], in which PV increase is a burden on cardiac tissue contraction for heart failure. Another highlight of our study is that the HFpEF subtype has a significant insulin‐induced in‐hospital death association in both the US and China. HFpEF often coexists with obesity and hypertension, manifested in abnormal PV and impaired venous capacitance [40]. The elevation of PV may be linked to the pathophysiology of HFpEF, including diastolic dysfunction and impaired ventricular filling, thereby reducing the cardiac reserve [41]. The impact of diuretic drug use is more pronounced in patients with HFpEF compared to other subtypes [42], suggesting a plausible diuretic treatment scheme and fluid restriction to control the adverse effects of insulin‐induced volume expansion.

One important clinical dilemma in managing PV in CHF‐T2DM patients was that diuretic drug use may relieve the symptoms of edema and dyspnea, but its long‐term use may lead to poor prognosis [43]. Furthermore, clinicians aimed to balance the need for glycemic control while preventing heart failure due to volume overload. The best strategy for diuretic regimens may be learned from practical histories [44, 45], along with investment in HF subtype‐specific hypoglycemic drugs [46], such as sodium glucose co‐transporter 2 [2], in achieving glycemic control while providing a diuretic response [20].

Our study presents strengths. First, we employed two EMRs with a matched cohort design which minimized potential confounding with LOS, CHF commonly used diuretics [47, 48], and commonly used oral drugs [49, 50] to adjust the model, which further improved the robustness of our results. Second, we introduced the ePVS as a surrogate measurement for the instantaneous blood volume in the past literature [24, 31]. Previous studies have revealed that elevated ePVS is associated with CHF's poor prognosis [51], but not in the case of insulin‐associated in‐hospital death.

Limitations should be considered when interpreting our findings. First, the retrospective nature of our EMRs may introduce recall bias. Second, a lack of time scale in our data limited the conclusion on the hazard ratio. Third, residual confounding may persist due to unmeasured lifestyle factors and missing BMI values that were not captured in our datasets. Fourth, our sample size for HF subtypes is uneven and thus might not be sufficiently powered.

## 5 Conclusions

Insulin therapy is associated with higher in‐hospital death in patients with CHF‐T2DM, which is mediated by increased PV. Clinicians should closely monitor PV changes in patients receiving insulin therapy.

## Author Contributions

G.F. and F.J. design and put forward the conceptualization. L.Z. and X.L. organized data curation, formal analysis, and writing – original draft. B.F. finished writing – review and editing. G.F. and L.Z. provides the funding acquisition and project administration. X.O. and J.L. promote resources, supervision, and validation. D.Z., H.G., and L.L. contributed to the interpretation of the results and investigation, methodology. M.X. and J.L. provides access and verification of the underlying data. All authors read and approved the final manuscript.

## Funding

This work was financially supported by the National Natural Science Foundation of China (No. 82430121) and China Postdoctoral Science Foundation (2025M773980).

## Ethics Statement

In TJHFIT, the ethics committee of First Teaching Hospital of Tianjin University of Traditional Chinese Medicine passed the ethics approval (TYLL2023[K]). The research has been registered (ChiCTR2300077220). In MIMIC, the author (Dr. Lin Zhang) was supported in extracting data from this database for the study (certification number record ID: 55394138).

## Consent

We have obtained permission from MIMIC and TJHFIT to publish the manuscript.

## Conflicts of Interest

The authors declare no conflicts of interest.

## Supporting information


**Table S1:** Absolute standardized mean differences between in‐hospital death and control before and after propensity score matching.
**Table S2:** The ICD code for type 2 diabetes mellitus.
**Table S3:** The ICD code for chronic heart failure.
**Table S4:** The subgroup analysis of insulin therapy and in‐hospital death in MIMIC.
**Table S5:** The subgroup analysis of insulin therapy and in‐hospital death in TJHFIT.
**Table S6:** Mediation analysis of ePVS.

## Data Availability

MIMIC data were derived from https://physionet.org/content/mimiciv/2.0/. The TJHFIT data can be obtained by contacting the corresponding author.
